# Royal Westminster Ophthalmic Hospital

**Published:** 1830-03

**Authors:** 


### HOSPITAL REPORTS.

### ROYAL WESTMINSTER OPHTHALMIC HOSPITAL.

*Report of the various Plans of Treatment pursued at this Hospital.* Drawn up by the direction of Mr. GUTHRIE, by J. FOOTE, jun.

### *Catarrhal Inflammation of the left Eye treated by copious Depletion. Six Pints of Blood taken in twenty-two Hours.*

ROBERT SAFE, ætat. forty, admitted September 13th, 1829, has been ill about ten or eleven days. He first attended about a week ago, when the inflammation was by no means so severe as at present. The Ung. Argent. Nitr. was applied, and he had six grains of the Hydrarg. Subm. to take at bedtime, and an ounce of the Magn. Sulph. in the morning.

The application of the ointment caused such pain, that it deterred him from attending again during the remainder of the week; though he himself said that he was more frightened than hurt, and that, when the irritation from the ointment had subsided, his eye was considerably better.

When he re-attended, considerable tumefaction of the lids existed, particularly of the lower one, which was also discoloured; the conjunctiva highly injected and swollen; a puriform discharge is also present, but not in great quantity, attended with severe pain round the orbit, increased so much at night that it prevents his sleeping. Has no appetite; much thirst; tongue white; pulse full, about ninety-eight.

He was seen at two in the afternoon, when venesection was performed to twenty-six ounces, and twenty ounces taken by cupping from the left temple. Habeat Hydr. Subm. gr. vi. h. s. sum. Magn. Sulph. ℥i. mane. Bathe the eye with cold lotions three or four times a day.

14th, ten o'clock A.M.—The pain was removed for a time by the bleeding, and he says he felt altogether better. It returned, however, during the night with as much violence as before. The appearance of the eye is much the same, though there is not so much inflammation. The discharge is the same, if any thing, rather thicker. Pulse about ninety-four, not so full; bowels open.—Venæsectio ad deliquium (℥xl.) Appl. Cucurb. cruent. ad ℥x. temp. sinist. ℞. Pulv. Ipec. c. gr. x.; Hyd. Subm. gr. iv. M. fiat pulv. h. s. s. Rep. Lotio frigid.

He was bled in the erect position, with a large opening, and in a full stream.

15th.—Fainted twice yesterday in consequence of the loss of blood. Is much better, and remains nearly free from pain. The discharge is less; the lids are not so much tumefied; can open his eye, and bear the light better.—Hyd. Subm. gr. vi. h. s. sum. Magn. Sulph. ℥i. mane. Rep. Lotio.

16th.—Mouth touched by the mercury. His eye is much better; the lids have nearly regained their natural appearance; the discharge is decreasing.—℞. P. Jalap. c. ʒi. mane sumend. Lotio Aluminis ocul.

17th.—The discharge has nearly ceased. Mouth still sore. Can see a great deal better; the lids have their natural appearance; the redness of the conjunctiva fast disappearing.—Repet. Pulv. et Lotio.

Has not since returned.—Case by J. Foote, jun.

*Inflammation of the Cornea with Ulcer after Smallpox, treated in the first instance by the Antim. Tart. unsuccessfully, and cured by the application of the Ung. Argent. Nitr.*

Betsy Froth, ætat. eighteen, admitted December 8th, 1829. Inflammation of the sclerotic and cornea, with ulcer on the cornea. Complains of great lachrymation and intolerance of light; very imperfect vision, accompanied by great pain in the eye and brow. The lids are much inflamed, and the sclerotic inflammation is very intense, forming a zone of thickly-set vessels round the cornea, which is clear except where the ulcer is situated, which is surrounded by an effusion of lymph. States that she was cured of the smallpox about a fortnight ago; since when, the present disease has been gradually appearing. When the disease first appeared, she made use of an empirical remedy, called the "golden ointment," which she thought did her good at the time, but it has now ceased to relieve her.—Appl. Cucurb. cruent. ad ℥xij. temp. ℞. Antimon. Tart. gr. ij.; Magn. Sulph. ℥ij.; Aquæ fervent. Oss. sum. 8vam partem omni horâ.

9th.—Is entirely free from pain. She thinks her vision is improved; the inflammation is diminished. The medicine made her sick last night, and during the retching blood flowed from the cupping; it has excited nausea and a sensation of fainting.—Cont. medicamenta.

10th.—Medicine causes vomiting night and morning. She is free from pain; vision is improved; the inflammation is diminished.—Cont. medic. sum. 8vam partem omni horâ et semisse.

11th.—Sees better, and was altogether better until four this morning, when she was attacked by a severe darting pain in the brow and eye, with increased lachrymation and intolerance of light. The sclerotic inflammation is more severe; the lids are highly inflamed. The ulcer is healing, and the vision is improved. The medicine does not cause vomiting at present, but continues to excite considerable nausea.—Appl. Hirudines iv. ad palp. infer. ℞. Antim. Tartar. gr. ij; Magnes. Sulph. ℥ij. Solve; sumatur 8vam partem omni horâ.

12th.—She complains of a severe darting pain in the brow, lasting about half an hour, and recurring once in the day. Vision remains much the same. The medicine continues to excite considerable nausea and purging, but all vomiting has ceased. Tongue white and furred. The inflammation is not so intense.—Appl. Hirud. iv. ad palp. Appl. Emp. Lyttæ pone aurem. Rep. Ant. Tartar. Antiphlogistic diet.

13th.—Is much better. The house-surgeon saw her, and directed three leeches only to the lower lid.—Rep. Ant. Tart.

14th.—The pain returned twice yesterday, but was neither so severe nor did it last so long. The sclerotic inflammation is more intense on the inner part of the ball, forming a complete network of vessels; lids inflamed. The ulcer and opacity of the cornea are diminishing in size. Great lachrymation evening and morning; tongue white and furred; tolerable appetite; pulse regular. Medicine excites nausea and purging. The blister rose well after the lapse of twelve hours.—Hirud. iv. ad palp. infer. Rep. medic.

15th.—Vision is a little improved since yesterday. Pain not so severe, nor does it recur so frequently; lachrymation and intolerance of light diminished; inflammation is likewise considerably better. The medicine excites much nausea.—Cont.

16th.—Is better.—Rep. Antim. Tartar. Omitte Hirudines.

17th.—Continued improving during the whole of the day of the 16th, but, owing to the non-application of the leeches, she became much worse in the course of the evening. The inflammation is more severe, and she complains of great pain in the eye. Vision much the same.—Rep. applic. Hirud. iv., et Mist. cum Antimon. Tartariz.

18th.—Is a great deal better; vision improved; less intolerance of light. Medicine still excites nausea.—Rep. Hirudines et Mist.

19th.—The inflammation is *again* much increased in severity; the sclerotic appears a complete network of pink vessels. Not much lachrymation; intolerance of light; vision unimproved; free from pain; lids highly inflamed. The ulcer appears much the same.—Appl. Ung. Nigr. ℞. Pulv. Jalap. c. ℈i. primo mane sumend.

21st.—Is much better; inflammation is abating.—Rep. Ung. et Pulv.

22d.—Is a great deal better; vision much improved; ulcer healing.—Rep. Ung. Nigr.

24th.—Inflammation disappearing; vision greatly improved.—Ung. Nigr.

26th.—The inflammation has again returned.—Appl. Ung. Arg. Nitr.

29th.—Much improved. Rep. Ung.

31st.—Vision is a little improved; free from pain.—Ung. rep.

January 2d, 1830.—Is a great deal better; the inflammation is fast disappearing, and vision is much improved; entirely free from pain.—Vin. Opii ad ocul.

5.—Nearly well; no inflammation.—Rep. Vin. Opii.

Case by J. Foote, jun.

*Inflammation of the Cornea with Ulcer, treated by Cupping and the Ung. Argent. Nitr.*

Elizabeth Green, ætat. twenty, admitted December 8th, 1829. Acute inflammation of the conjunctiva and cornea, with ulceration.

Complains of great pain in the brow, and a feeling as if sand were under the lid; vision nearly lost. Catamenia not regular; tongue white; bowels costive. Her eye has been inflamed three weeks; thinks it proceeds from cold.—Appl. Cucurb. cruent. ad ℥x. temp. Appl. Ung. Argent. Nitr. ad ocul. ℞. Hyd. Subm. gr. vi. h. s. s. Magn. Sulph. ℥i. mane sumend.

12th.—Is much better; the pain is much relieved, and is situated more in the brow at present. The inflammation is still very great; not much lachrymation. Vision much improved.—Rep. Ung. Nigr. Cont. Pil., Magn. Sulph.

13th.—Is better. Rep. Ung.

15th.—Is a great deal better; ulcer healing; not so much lachrymation; the inflammation is abating; does not suffer so much pain as before; vision much improved.—Rep. Ung.

17th.—Ulcer healing; inflammation diminished.—Rep. Ung.

19th.—Inflammation nearly gone; vision much improved.—Rep. Ung.

22d.—Nearly well. Rep.

24th.—Inflammation has disappeared. Rep. Ung.

29th.—Ulcer healed.—Appl. Vin. Opii pro leucomâ.

Case by J. Foote, jun.

*Inflammation of the Cornea, treated by the Ung. Argent. Nitr.*

Sarah Ashley, ætat. fifteen, admitted October 29th, 1829, with inflammation of the cornea of each eye: both cornea much thickened and muddy; the right is much the worst; vision nearly lost. Complains of pain in the head. Bowels constipated; tongue clean; pulse natural. The inflammation commenced about six months ago.—Appl. Ung. Nigr. ℞. Ext. Coloc. c. gr. iij.; Hyd. Subm. gr. j. M. fiat pil. horâ somni sumend. ℞. Sulph. Magn. ℥i. mane primo sumend.

31st.—Feels better since the application of the ointment, and wishes it to be repeated.—Appl. Ung. Nigr.

November 3d.—The inflammation of the cornea of the left eye is almost removed; the muddy appearance of the right is much diminished.—Rep. Ung. Pilul. purg. omni nocte.

5th.—Is much improved.

14th.—The inflammation of the left cornea has disappeared, and the right is much improved. Complains of very little pain in the head. Bowels costive; pulse natural.—℞. Pulv. Jalap. c. ℈i. nocte. Magn. Sulph. ℥i. mane.

17th.—Cont. medic.

24th.—Is very much improved. Rep. Ung.

December 1st.—The muddy appearance is nearly removed.—Rep. Ung.

15th.—The right eye continues improving, and her vision is nearly as good as ever.—Appl. Ung. Nigr. Rep. Pulv. purg. et Sulph. Magn.

28th.—The muddy appearance is entirely removed; the inflammation is very slight, and merely of the lids; vision as good as ever.—Ung. rep.

Discharged cured.—Case by Mr. Clark, M.R.C.S. Edin.

